# Key steps of complex robotic liver surgery: an international expert survey

**DOI:** 10.1007/s00464-025-12020-9

**Published:** 2025-08-21

**Authors:** Noa L. E. Aegerter, Christoph Kuemmerli, Felix Nickel, Cristiano Guidetti, Christoph Tschuor, Victor Lopez-Lopez, Taiga Wakabayashi, Philipp Dutkowski, Adrian T. Billeter, Beat P. Müller, Philip C. Müller, Complex Robotic Liver Surgery Group, Yuta Abe, Mohammad Abu Hilal, Jawad Ahmad, Luca Aldrighetti, Adnan Alseidi, Ugo Boggi, Dieter C. Bröring, Hop Tran Cao, Yee Lee Cheah, Roland Croner, Fabrizio Di Benedetto, Alessandro Ferrero, David Geller, Stefan Gilg, Brian K. P. Goh, Jeroen Hagendoorn, Jason Hawksworth, Jin He, Asmus Heumann, Mathieu D’Hondt, Jan Philipp Jonas, Yoshikuni Kawaguchi, Philipp Kron, Jae Hoon Lee, Mickael Lesurtel, Chetana Lim, Charles Chung-Wei Lin, Georg Lurje, Marcel Machado, John B. Martinie, Riccardo Memeo, Yutaka Nakano, Christian E. Oberkofler, Fabrizio Panaro, James Park, Hugo Pinto Marques, Johann Pratschke, Florian Primavesi, Nuh Rahbari, Francesca Ratti, Christoph Reissfelder, Ricardo Robles-Campos, Olivier Saint-Marc, Olivier Scatton, Moritz Schmelzle, Daniel Seehofer, Olivier Soubrane, Patrick Starlinger, Stefan Stättner, Benjamin Strücker, Iswanto Sucandy, Roberto I. Sutcliffe, Rutger-Jan Swijnenburg, Christian Toso, Roberto I. Troisi, Go Wakabayashi, Roeland de Wilde

**Affiliations:** 1 Clarunis, University Digestive Health Care Centre Basel, Basel, Switzerland; 2 Department of Surgery, Erasmus MC Cancer Institute, Rotterdam, The Netherlands; 3 Klinik und Poliklinik für Allgemein-, Viszeral- und Thoraxchirurgie, Universitätsklinikum Hamburg-Eppendorf, Hamburg, Deutschland; 4 Hepato-Pancreato-Biliary Surgery and Liver Transplantation Unit, University of Modena and Reggio Emilia, Modena, Italy; 5 Department of Surgical Gastroenterology and Transplantation, Rigshospitalet, Copenhagen University Hospital, Copenhagen, Denmark; 6 Department General Surgery and Abdominal Solid Organ Transplantation Unit, University Clinical Hospital Virgen de La Arrixaca, IMIB-Pascual Parrilla, Murcia, Spain; 7 Department of Surgery, Keio University School of Medicine, Tokyo, Japan; 8 Department of Surgery, Ageo Central General Hospital, Ageo, Japan

**Keywords:** Robotic liver surgery, Liver resection, Complex robotic liver resection

## Abstract

**Background:**

Robotic liver surgery (RLS) has become the preferred minimally invasive approach for liver surgery. However, especially for complex RLS (C-RLS), key surgical steps such as preoperative preparation, intraoperative techniques, and training are often center-dependent and not standardized. The aim of this survey was to assess the international practice of key surgical steps during C-RLS among expert centers.

**Methods:**

A cross-sectional survey was conducted among robotic liver surgeons with a minimum individual experience of 50 RLS to assess their practice during C-RLS. The survey consisted of 50 questions, distributed across three sections: training, preoperative planning, and intraoperative practice for C-RLS.

**Results:**

60 out of 71 experts completed the survey, corresponding to an 85% response rate. 73% of the experts agreed that the IWATE difficulty score represents an adequate classification system to define C-RLS. A prerequisite before performing C-RLS was experience in complex open liver surgery (71%) and expertise in low and intermediate RLS (75%). Mentoring by a more experienced surgeon was deemed necessary by most experts (90%) when performing C-RLS. Vascular inflow control was mentioned to often be performed during parenchyma transection either selectively (38%) or routinely (52%). Most experts considered pre- or intraoperative positive staining helpful (57%), while negative staining (85%) was reported as even more important in C-RLS. For vasculo-biliary transection, experts preferred an intrafascial (45%), glissonian pedicle approach (33%) or a case-dependent transection (12%). For parenchymal transection, the preferred instruments were laparoscopic CUSA (92%), harmonic ACE (78%), and SynchroSeal (77%).

**Conclusion:**

This expert survey reveals current international practices for preoperative preparation, training, and intraoperative key steps of C-RLS. Prospective validation of the key steps would be useful for correlating clinical outcomes with current practice.

The introduction of minimally invasive liver surgery (MILS) has been a key technological advancement in hepato-biliary surgery over the last two decades. Expert centers have rapidly adopted MILS, and especially robotic liver surgery (RLS) has started to become the preferred minimally invasive approach for many indications [1–3].

Due to the stable platform, the improved 3D vision, the possibility of depicting biliary structures with indocyanine green and the seven degrees of freedom of the robotic instruments, RLS is easier to perform, especially in complex cases, with lower conversion rates than the laparoscopic approach, while providing the same benefits of the minimally invasive access as laparoscopic surgery [4, 5].

Compared to open liver surgery, MILS offers an improved perioperative risk profile, a reduced risk of decompensation in cases of cirrhosis, and a shorter learning curve [6, 7]. Recently, complex RLS (C-RLS), such as hemihepatectomies, two-stage hepatectomies, perihilar cholangiocarcinoma resections, living donor liver transplantation, and full graft liver implantation, including vasculo-biliary reconstructions, have been adopted by international expert centers [8–11].

However, even after two recent consensus meetings in Southampton and Paris, key surgical steps, such as for preoperative preparation, intraoperative techniques, and training for C-RLS, are often center-dependent and not standardized [12, 13]. While international guidelines recognize MILS as the standard approach for minor liver resections, C-RLS has not been explicitly addressed in those consensus meetings [12–14].

The aim of this expert survey was to assess the international practice of key surgical steps, including robotic training, preoperative preparation, intraoperative imaging, and surgical techniques in C-RLS.

## Materials and methods

### Expert panel

International experts were invited based on their institutional and individual surgical experience in RLS, including a documented interest in academic output on MILS. The inclusion criteria for invited experts were 1) Specialization in hepato-biliary surgery with a minimum personal experience of 50 RLS. 2) Institutional caseload of a minimum of 25 major liver resections/year and 30 RLS/year. Experts were contacted via email, which included a link to the survey and the anticipated time commitment.

### Definition of complex robotic liver surgery

Definitions for C-RLS differ and several difficulty scoring systems for MILS and RLS have been proposed [15–19]. Since the IWATE difficulty scoring system is the most frequently used scoring system for MILS, C-RLS was defined as resections with an IWATE score from 7 to 12 for the current survey. Those resections correspond to an advanced or expert difficulty level, meaning that C-RLS stands for right or left hepatectomies or posterior sectionectomies with large tumors ($\ge$ 3 cm) close to large vessels [15].

### Survey design

**Fig. 1:**
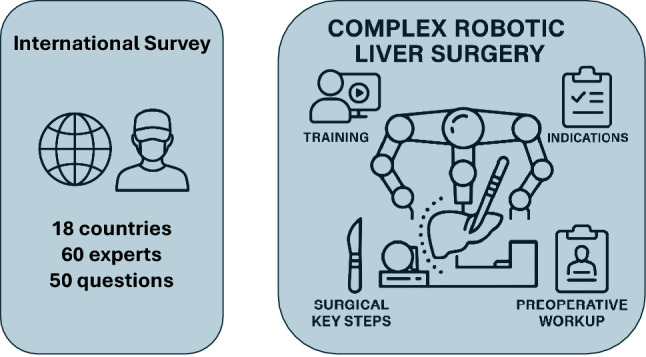
Visual abstract of the survey on key steps of complex robotic liver surgery

As a first step, the steering committee (NLEA, CK, FN, CG, CT, VLL, TW, ATB, PCM) reviewed the literature on major and technically major RLS to assess current surgical standards for preoperative planning, surgical key steps, and important aspects of training for RLS. In a second step, the steering committee drafted an online survey and held two online meetings to adapt the questionnaire. After two rounds of revisions by the steering committee, the online survey was sent to the expert group. The survey was designed with SurveyMonkey Inc., San Mateo, California, USA. (www.surveymonkey.com). It consisted of 50 open-ended questions on surgeon- and center-specific practices on training for C-RLS, preoperative planning, and intraoperative key steps (Fig. 1). Experts were given 4 weeks to complete the survey. Two reminders were sent, the first 2 weeks after opening and the second 1 week before closing. This survey is reported in accordance with the Checklist for Reporting of Survey Studies (CROSS) [20].

Data collection took place from February 1, 2025, to February 28, 2025. Double participation or answers after the inclusion period were excluded. The answers of incomplete surveys were included.

### Data analysis

The experts’ answers were analyzed using descriptive statistics. For continuous variables, the median and interquartile range (IQR) are reported, using the Mann–Whitney U test. Categorical variables are reported as counts and percentages using the Fisher’s exact test. Statistical Analysis was performed with R (R Core Team (2024). R: A language and environment for statistical computing. R Foundation for Statistical Computing, Vienna, Austria. URL https://www.R-project.org/, version 4.4.2).

## Results

### Expert panel

**Fig. 2:**
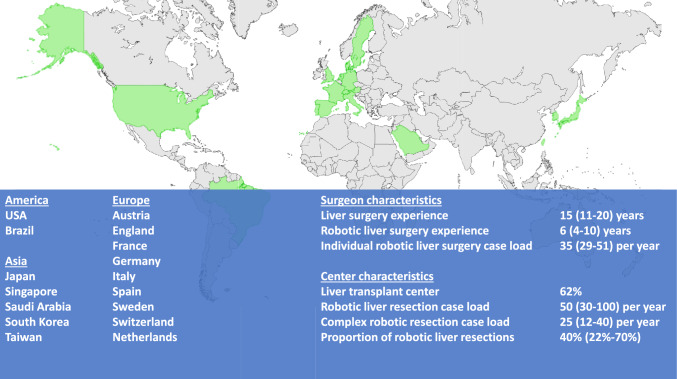
Summary of participating countries, center-specific case load and expert experience

Seventy-one experts were invited, of whom 60 completed the survey, corresponding to an 85% response rate. Four respondents completed the survey partially. Experts from 18 countries, spanning three continents—Asia (15%), Europe (67%), and North and South America (18%)—participated. All participants were specialist surgeons with an annual experience of 50 (range 30–100) robotic liver resections, which accounts for 40% (22–70%) of all liver resections performed at their institutions. The institutional and individual characteristics of the participating experts are presented in Fig. 2.

## Survey

### Definition of complex robotic liver surgery

Forty-four of the experts (73%) agreed that the IWATE difficulty score represents an adequate classification system to define C-RLS. Others suggested using the French difficulty score or the Kawaguchi three-level complexity classification instead [17, 21]. Some experts mentioned specific features that should be incorporated in a more adequate difficulty scoring for C-RLS, alternatively: obesity (*n* = 3), previous oncologic treatments like chemotherapy, local ablation, or surgery (*n* = 3), vascular and biliary reconstructions (*n* = 1), and liver parenchyma quality (*n* = 1). The complete survey results are presented in Table 1.

**Table 1:** Survey results

	Overall (*n* = 60)
Contraindications for C-RLS	
Biliary reconstruction	9 (15)
Vascular resection	17 (28)
Vascular reconstruction	36 (60)
Previous liver or complex upper GI surgery	4 (6.7)
Two-stage approach (e.g., ALPPS)	14 (23)
Perihilar CC	22 (37)
Other	12 (20)
Patient positioning	
Standard for tumors in the right hemi liver	
Left lateral decubitus	11 (18)
Modified French position	35 (58)
Other	12 (20)
Standard for tumors in the left hemi liver	
Left lateral decubitus	3 (5)
Modified French position	40 (67)
Other	15 (25)
Pringle maneuver	
Selective	23 (38)
Standard	31 (52)
External	19 (32)
Internal	38 (63)
Is preparation of Pringle maneuver necessary before parenchymal transection?	
Yes	52 (88)
No	4 (6.7)
IWATE	
Is the IWATE classification adequate for the definition of C-RLS	
Yes	44 (73)
No	13 (22)
Training	
Do you have a defined training program for surgeons for C-RLS?	
Yes	21 (35)
No	36 (60)
Does the program have to be completed before performing C-RLS?	
Yes	32 (53)
No	15 (25)
Other training before C-RLS	9 (15)
Is mentoring required before performing C-RLS?	
Yes	54 (90)
No	1 (1.7)
Is dual-console training part of the C-RLS training?	
Yes	37 (62)
No	17 (28)
It should be mandatory to have experience in complex open LS	43 (72)
It should be mandatory to have experience in complex laparoscopic LS	16 (27)
It should be mandatory to have experience in low/intermediate RLS	45 (75)
Do you have standardized criteria that have to be fulfilled before performing C-RLS?	
Yes	
No	20 (33)
Insufflator	
What kind of inflator devices should be used for C-RLS?	
AirSeal	14 (23)
Standard laparoscopic/robotic trocar	33 (55)
Other	11 (18)
Imaging	
Is specific imaging necessary before C-RLS?	
Yes	51 (85)
No	6 (10)
What kind of imaging is necessary?	
CT	21 (35)
MRI	13 (22)
Other	20 (33)
Would 3D reconstructed, intraoperatively available image be beneficial?	
Yes	51 (85)
No	6 (10)
Workup	
Does liver-specific workup differ for C-RLS compared to the open approach?	
Yes	18 (30)
No	38 (63)
Assistance	
Should another senior HPB surgeon assist during C-RLS?	
Yes	36 (60)
No	20 (33)
How many assistance ports should be available in C-RLS?	
1	20 (33)
1–2	14 (23)
1–3	1 (1.7)
2	18 (30)
3	2 (3.3)
Instruments for C-RLS	
Are specific instruments (robotic bulldogs/clamps) mandatory for C-RLS?	
Yes	49 (82)
No	8 (13)
Ultrasound	
Is intraoperative ultrasound needed for C-RLS?	
Yes	56 (93)
No	1 (1.7)
Staining	
Is preoperative/intraoperative positive staining helpful in C-RLS?	
Yes	34 (57)
No	22 (37)
Is preoperative/intraoperative negative staining helpful in C-RLS	
Yes	51 (85)
No	6 (10)
Which method do you use	
Both	34 (57)
Intraoperative staining for vascular demarcation	19 (32)
Preoperative staining for tumor identification	3 (5)
Vascular/biliary transection	
Preferred approach for transection of vascular/biliary structures in C-RLS	
Conventional intrafascial approach	27 (45)
Glissonian pedicle approach	20 (33)
Other	10 (17)
Conversion	
Should instruments for a conversion to a laparoscopic approach be present in the OR?	
Yes	27 (45)
No	29 (48)
Should instruments for a conversion to an open approach be present in the OR?	
Yes	48 (80)
No	8 (13)

*CC* Cholangiocarcinoma, *C-RLS* complex robotic liver surgery, *GI* Gastrointestinal, *LS* Liver surgery, *RLS* Robotic liver surgery

### Contraindications for C-RLS

The most common institutional contraindications for C-RLS were vascular reconstructions (*n* = 36, 60%), surgery for perihilar cholangiocarcinoma (*n* = 22, 37%), vascular resection (*n* = 17, 28%), and two-stage hepatectomies (*n* = 14, 23%). Other contraindications included large tumor lesions (*n* = 3), tumors in close contact with the hepatic vein confluence or portal bifurcation (*n* = 3), and the need to perform multiple metastasectomies in both hemi-livers (*n* = 1).

### Training for C-RLS

Most centers did not have a particular program to train surgeons specifically for C-RLS (*n* = 36, 60%). However, almost all participants (*n* = 54, 90%) agreed that mentoring by a more experienced surgeon was necessary when performing C-RLS, and dual-console training (*n* = 37, 62%) was considered an adequate method to achieve this. Furthermore, experts agreed that before performing C-RLS experience in complex open liver surgery should be mandatory (*n* = 43, 71%) alongside expertise in low and intermediate RLS (*n* = 45, 75%). The suggested previous experience of robotic liver surgeries ranged from 10 to 150. Interestingly, only a minority of experts mentioned complex laparoscopic liver surgery as a prerequisite for C-RLS (*n* = 16, 27%).

### Preoperative workup

Most experts suggested a similar workup for C-RLS as for the open approach (*n* = 38, 63%). Some mentioned differences in the preoperative workup compared to the open approach, including a briefing with anesthesia about the risk of conversion (*n* = 2) and case duration (*n* = 1).

Furthermore, preoperative ICG administration (*n* = 3) and volumetric reconstruction (*n* = 2) were mentioned as specific preoperative differences. Finally, an overall more well-coordinated team, including surgeons, scrub nurses, and anesthesia, was suggested (*n* = 3). With the importance of adequate preoperative imaging in MILS, experts preferred a CT scan (*n* = 21, 35%), an MRI scan (*n* = 13, 22%), or both (*n* = 11, 19%). A majority of experts (*n* = 51, 85%) would find an intraoperatively available 3D reconstruction of the preoperative imaging beneficial.

### Surgical setup

For C-RLS, table assistance by a senior HPB surgeon was mandatory for the majority (*n* = 36, 60%). Most experts reported the modified French position as their preferred patient positioning in cases of tumors in the right (*n* = 36, 60%) and left liver (*n* = 40, 67%). On the other hand, a minority preferred the supine position for tumors in the right (*n* = 8, 13%) and left liver (*n* = 11, 18%).

For the pneumoperitoneum, a standard laparoscopic or robotic trocar (*n* = 33, 55%), the AirSeal device (*n* = 14, 23%), or the Lexion device (*n* = 2, 2%) is used. Most experts use one or two assistance ports for C-RLS (1 port: *n* = 20, 33.3%; 1–2 ports: *n* = 14, 23.3%; 2 ports: *n* = 18, 30%). Almost all experts (*n* = 49, 82%) agreed that specific instruments are needed for C-RLS, especially robotic bulldogs or robotic clamps for vascular control.

### Intraoperative key steps

A Pringle maneuver for vascular inflow control is often prepared in C-RLS, either for selective (*n* = 23, 38%) or routine inflow occlusion (*n* = 31, 52%) during parenchyma transection. Two-thirds of experts use an internal Pringle maneuver (*n* = 38, 63%), and one-third use an external Pringle maneuver (*n* = 19, 32%). Almost all experts agreed that intraoperative ultrasound (IOUS) is necessary during C-RLS (*n* = 56, 93%), especially before parenchymal transection and during the break of inflow occlusion.

Most experts considered pre- or intraoperative positive staining helpful in C-RLS (*n* = 34, 57%), however, negative staining (*n* = 51, 85%) was reported as even more important in C-RLS. With regard to vasculo-biliary transection, nearly half of the experts (*n* = 27, 45%) prefer a conventional intrafascial approach, while one-third use a glissonian pedicle approach (*n* = 20, 33%). The remaining experts (*n* = 7, 12%) selectively apply both approaches depending on the specific case of C-RLS. The most commonly used devices for a standard liver parenchymal transection were the laparoscopic CUSA (*n* = 51, 85%), followed by the harmonic ACE (*n* = 46, 77%), the SynchroSeal (*n* = 45, 75%), and the Vessel Sealer (*n* = 44, 73%). This did not significantly change for other scenarios of major C-RLS, including cirrhotic or steatotic parenchymal transections, as displayed in Table 2.

**Table 2:** Use of transection devices for robotic liver resections

	Major resection	Normal parenchyma	Cirrhotic/steatotic parenchyma
Laparoscopic CUSA	50 (83)	51 (85)	51 (85)
Vessel sealer	42 (70)	44 (73)	46 (77)
SynchroSeal	44 (73)	45 (75)	44 (73)
Harmonic ACE	47 (78)	46 (77)	45 (75)
Bipolar forceps	18 (30)	20 (33)	16 (27)
Monopolar scissor	42 (70)	41 (68)	39 (65)

Considering conversion, half of the surgeons have the necessary instruments for a potential conversion of C-RLS ready in the operating room (*n* = 27, 45%). The experts also mentioned that not only should the surgical staff be trained for a possible emergency conversion, but a possible conversion should also be discussed before the start of each C-RLS case. Some experts additionally recommended that a backup HPB surgeon should be available on standby.

## Discussion

The current literature on C-RLS is scarce, and little is known about international practice standards, as the literature mainly focuses on minor robotic liver resections. As such, the Southampton Guidelines included two sections on the role of RLS, noting that it provides similar outcomes to laparoscopic resections, albeit with longer operative times and higher costs. However, the topic of C-RLS was not specifically covered [12]. Likewise, the Paris Consensus Conference on robotic Hepato-Pancreato-Biliary (HPB) Surgery focused on technology, training, expertise, and outcome assessment of robotic HPB surgery [13]. Again, the focus was on minor RLS and did not address the topic of C-RLS [13]. Therefore, this survey is much needed to provide insight for the rapidly evolving field of complex and major RLS.

The findings of this international expert survey provide key insights into training, preoperative planning, and key intraoperative steps for C-RLS among 60 experts in RLS. This large survey highlights the need for adequate training before performing C-RLS, as well as the importance of pre- and intraoperative imaging to compensate for the loss of tactile feedback. Furthermore, experts emphasized the importance of safety maneuvers, including inflow control, positive and negative staining of the liver remnant, and the availability of an emergency conversion concept in the event of major robotic resections.

To define C-RLS, several scoring systems have been proposed in the past. However, most of them were primarily initiated and validated to assess the complexity of laparoscopic liver surgery. In clinical practice and for research purposes, the most frequently used are the IWATE, Institut Mutaliste Montsouris (IMM), Southampton, and Hasegawa difficulty scores [15, 22, 23]. According to a recent systematic review, all four difficulty scoring systems (DSS) correlated with intra- and postoperative outcomes for laparoscopic liver surgery [23]. With the emergence of robotic liver surgery, the DSS has also been validated for RLS. The different DSS had variable strengths and weaknesses in predicting conversion or postoperative complications [24]. The team from Tampa recently proposed the Tampa Difficulty Score, which was developed specifically for RLS [24]. Still, international expert guidelines on RLS recommend using the Ban and IWATE systems as they are the most validated DSS for RLS [14]. This aligns with the results of the current survey, in which most experts supported the IWATE DSS to classify the complexity of RLS. Some specific adoptions may further enhance the accuracy of the IWATE system, as vascular and biliary resections and reconstructions are increasingly performed using the robotic approach and should be incorporated into a revised DSS system. Furthermore, RLS might make some difficult-to-reach segments (posterosuperior) with laparoscopy more approachable, and in general, complex resections more feasible with RLS [25]. This is reflected in the fact that vascular resections, two-stage hepatectomies, and even Klatskin resections were no longer considered strict contraindications for RLS at the included expert centers.

Most centers recognize the importance of adequate training before embarking on C-RLS. However, structured training programs for C-RLS such as the LIVEROBOT curriculum from Amsterdam were only available in 40% of all participating centers. Currently, most surgeons’ learning curve consists of open, laparoscopic, and minor robotic liver resections before embarking on C-RLS.

Studies examining the learning process in RLS emphasize the importance of a comprehensive learning curve assessment, encompassing both minor and major hepatectomies, as well as various numbers to achieve improvements in intraoperative and postoperative outcomes. In a recent large systematic review of learning curves in minimally invasive liver surgery, improvements in intraoperative outcomes were observed first (*competency phase*), while significantly more experience was required to improve postoperative complications and oncologic outcomes (*proficiency and mastery phases*) [6].

This proposed standardization of a three-phase model is supported by findings from a specialized center in Denmark, where a minimum of 30 low-to-moderate difficulty robotic procedures are recommended before proceeding to more complex resections [26]. Following minor hepatectomies, a learning curve of 40–60 cases is recommended to achieve proficiency for robotic major right lobe segmentectomy. Similarly, to overcome the three phases of the learning curve for major robotic hepatectomy, a total of 92 patients were needed—15 patients for the initial learning phase, 25 for the intermediate phase, and 52 to achieve mastery [27]. In contrast, recent consensus guidelines recommend only 15 cases for minor RLS and 25 cases of major RLS for an experienced surgeon to overcome the learning curve [14]. However, this strongly depends on previous experience in open and laparoscopic liver surgery, which was also emphasized in this survey. For example, D’Hondt et al. investigated the transition from a laparoscopic to a robotic liver surgery program [25]. With extensive experience in LLS, the surgeon was able to rapidly overcome the learning curve for RLS [25]. Even more outstanding, the short-term outcomes of the implementation phase of RLS were comparable to those of the mastery phase of LLS, indicating that even more complex resections may be attempted with the robotic approach once the learning phase for RLS is complete [25]. For institutions initiating a robotic liver surgery program, we strongly recommend following a structured program, such as LIVEROBOT (https://precisionhpbsurgery.com), with mentoring from international tutors who have completed the personal and institutional learning curve. While previous experience in laparoscopic liver surgery seems to be of less importance, around 20–30 minor robotic resections (IWATE score 0–6) should be performed before C-RLS. In line with this, surgeons should be aware of their current position on the learning curve and adjust case complexity according to validated DSS, while comparing postoperative outcomes to international benchmark values [3].

Regarding specific measures for C-RLS, the majority of experts agreed that high-quality imaging is necessary, and most surgeons would like to have three-dimensional imaging available intraoperatively during C-RLS. Other specific measures included a more detailed case discussion with the anesthesia team and scrub nurses, focusing on prolonged operation times and strategies for emergency conversions.

Another critical aspect of implementing RLS is the lack of a robotic CUSA device, the preferred transection device in open and laparoscopic liver surgery [28]. Parenchymal transection during RLS can be performed with specific instruments (e.g., Vessel Sealer, SynchroSeal) or the available monopolar scissors or bipolar grasper [29]. As shown in our survey, many surgeons still use the laparoscopic CUSA in case of C-RLS, followed by the harmonic ACE, the SychroSeal, and the Vessel Sealer. Their choice of the parenchymal transection tool did not differ significantly between normal and cirrhotic liver parenchyma. The choice of the laparoscopic CUSA may further influence the need for a trained HPB surgeon at the table, a suggestion made by 60% of the experts. An alternative to the laparoscopic CUSA presents the double bipolar forceps-clamp-crush technique. The two techniques were compared in a large, propensity-matched, multicenter study involving 1,070 consecutive robotic liver resections. No significant differences were found in blood loss, operative time, and postoperative complications [30]. Another study compared the bipolar Vessel Sealer with the newly developed SynchroSeal in 300 patients undergoing liver resection [31]. After propensity matching, the SynchroSeal showed reduced intraoperative blood loss (95 mL versus 48 mL), while all other postoperative outcomes were comparable [31]. Currently, several transection devices are available that allow for safe and bloodless parenchymal transection. As in open liver surgery, the preferred device is mainly dependent on the surgeon.

This study has some limitations. The study was conducted as a survey, and data can be biased or inaccurate, as they depend on the personal opinion of the corresponding expert. Therefore, not all current practices might be displayed. Furthermore, while experts from all over the world were invited to participate in the survey, two-thirds of the respondents were from Europe, indicating that current practices from Asia and America may be underrepresented. However, compared to previous studies, this is by far the most extensive survey on robotic liver surgery. As a third limitation, the survey was designed with predetermined questions, which may have limited the topics and practices gathered during the survey. However, at the end of every question, experts had the option to provide their feedback in free-text form.

In conclusion, the findings from this international expert survey display current practices during the preparation, training, and surgery of C-RLS. While preoperative preparation is mainly standardized and does not significantly differ from open or laparoscopic surgery, key surgical steps are non-standardized, and especially the strategies to prepare the vasculo-biliary structures and parenchyma transection vary among experts. Further studies should aim to prospectively validate the key steps during C-RLS and correlate clinical outcomes with current practice.

## Abbreviations

C-RLS: Complex Robotic Liver Surgery
DSS: Difficulty Scoring Systems
EBL: Estimated Blood Loss
IQR: Interquartile Range
LOS: Length of Stay
MILS: Minimally invasive Liver Surgery
RLS: Robotic Liver Surgery
